# Detection and analysis of graduate students’ academic emotions in the online academic forum based on text mining with a deep learning approach

**DOI:** 10.3389/fpsyg.2023.1107080

**Published:** 2023-04-20

**Authors:** Qiaoyun Xu, Sijing Chen, Yan Xu, Chao Ma

**Affiliations:** ^1^Normal School, Jinhua Polytechnic, Jinhua, China; ^2^National Engineering Research Center for Educational Big Data, Central China Normal University, Wuhan, China; ^3^School of Marxism, Shanghai University of Finance and Economics, Shanghai, China; ^4^College of Economics and Management, Zhejiang Normal University, Jinhua, China; ^5^Institute of Scientific and Technical Information of China, Beijing, Beijing, China

**Keywords:** academic emotion, emotion recognition, emotion classification, graduate mental health, deep learning

## Abstract

**Purpose:**

The possibility of mental illness caused by the academic emotions and academic pressure of graduate students has received widespread attention. Discovering hidden academic emotions by mining graduate students’ speeches in social networks has strong practical significance for the mental state discovery of graduate students.

**Design/methodology/approach:**

Through data collected from online academic forum, a text based BiGRU-Attention model was conducted to achieve academic emotion recognition and classification, and a keyword statistics and topic analysis was performed for topic discussion among graduate posts.

**Findings:**

Female graduate students post more than male students, and graduates majoring in chemistry post the most. Using the BiGRU-Attention model to identify and classify academic emotions has a performance with precision, recall and F1 score of more than 95%, the category of PA (Positive Activating) has the best classification performance. Through the analysis of post topics and keywords, the academic emotions of graduates mainly come from academic pressure, interpersonal relationships and career related.

**Originality:**

A BiGRU-Attention model based on deep learning method is proposed to combine classical academic emotion classification and categories to achieve a text academic emotion recognition method based on user generated content.

## 1. Introduction

Graduate education is an important part of higher education. In the pursing of a master or doctoral degree, graduate students have been actively carrying out scientific research achievements. They take advantage of their intelligence and make the best use of higher quality scientific and technological resources. At the same time, they are also facing great pressure (Scott and Takarangi, 2019). Graduate students’ mental health concerns have been discussing for nearly 10 years and have attracted more and more attention (Gewin, 2012). These achievements and stress are expressed in many ways, of which emotions are mostly significant. Emotions have a great influence on human learning, memory, motivation, mental health, and neurological function. Positive emotions can increase the level of dopamine in the brain, which in turn affects the function of memory, learning attention, and the ability of creative problem solving (Ashby and Isen, 1999; Meinhardt and Pekrun, 2003; Ainley et al., 2005), meanwhile, negative emotions will reduce learning motivation and effort. Pekrun et al. (2002) first clearly put forward the concept of academic emotion in early 21st. Academic emotions refer to various emotional experiences related to students’ academic work in the teaching or learning process.

However, emotions are not easy to identify sometime. We cannot just ask some other graduate students with serial rude and personal questions such as “Are you happy doing your research today?” or “Why are you so angry about your teammates?” (Gratz and Roemer, 2004). So, if we want to discover the hidden emotions of graduate students, we first need them to be willing to express their emotions. With the continuous development of network technology, various social platforms have become centers for discussions and opinions (Sobkowicz et al., 2012). In such an anonymous environment, topics are being discussed, opinions are being published, and emotions are being expressed. Meanwhile, opinion mining methods based on machine learning and deep learning technologies have been widely accepted (Ramakrishnan et al., 2020), the task of sentiment analysis and emotion recognition for large-scale data sets has become feasible (Sánchez-Núñez et al., 2020). Thus, performing a data mining on the topics and comments published by these graduate students is a worthwhile path to discover their emotion states and care about their mental health, and our research mainly focuses on the following research questions:

1. What is the current state and distribution of graduate students who publish information on social platforms?
2. Based on these students’ posts, how can we discover the hidden academic emotions?
3. What are these graduate students really talking about online?

To address these issues, we conducted the following research. We performed our research on real Internet user generate contents. First, we conduct a descriptive statistical analysis on the collected data, to find out the distribution of posters’ information. Second, we transformed the task of discovering academic emotions from posters’ topics and replies into a supervised deep learning based classification model with natural language processing methods to recognize and classified the content into several emotion categories. At last, a semantic based topic evolution analysis has been conducted to find out what are the posters really discussing about specifically.

## 2. Related studies

### 2.1. Academic emotion recognition and measurement

Academic emotion recognition can be considered as the process of discovering hidden emotions from data and research in the studying process of students. Linnenbrink and Pintrich (2002) ran a dichotomy between positive and negative valence as an early exploration on academic emotion recognition and classification. From the research method and technology aspect, three types of academic emotion studies are widely conducted: empirical studies based on qualitative or quantitative questionnaires, model-based academic emotion recognition and measurement, and case studies based on meta-analysis. The Achievement Emotions Questionnaire (AEQ) is an important foundational questionnaire structure for the first type if studies. Pekrun et al. (2011) conducted AEQ and classified academic emotion into 9 categories as enjoyment, hope, pride, relief, anger, anxiety, shame, hopelessness, and boredom from three aspects as learning-related emotions, class-related emotions, and exam-related emotions. Based on these categories, researchers have expanded the applicable field of AEQ and proposed more new dimensions such as AEQ-S (Bieleke et al., 2021), AEQ-ES (Lichtenfeld et al., 2012) and AEQ-PE (Fierro-Suero et al., 2020). The second type of studies focuses on the optimization of academic emotion models. These studies approached on the construction of academic emotional models, such as OCC model (Clore and Ortony, 2013), categorical emotion models (D'mello and Graesser, 2007; Sreeja and Mahalaksmi, 2015) and dimensional emotion models (Sreeja and Mahalakshmi, 2017) are helpful for more accurate and comprehensive descriptions of the emotional state and can provide some references for the development of research work related to emotion recognition. The third type of studies is based on different cases, using meta-analysis method to comprehensively analyze the influencing factors of academic emotions. MacCann et al. (2020) discussed the relationship between emotional intelligence and academic performance. Camacho-Morles et al. (2021) analyzed the how could activity achievement emotions influence on academic achievement.

Recent studies have been conducted primarily on specific groups or by incorporating specific environmental factors. Wang et al. (2017) discussed the relationship between academic emotion and psychological well-being among Chinese rural-to-urban migrant adolescents. Wu et al. (2022) used questionnaire survey to study whether taking important exams during the COVID-19 pandemic would have an impact on students’ academic emotion and found that there was a certain negative deactivate correlation. Fried et al. (2022) ran an assessment of mental health, academic emotion, and resilience among undergraduate and graduate students, found that graduate students generally feel more stressed about their studies.

Meanwhile, the research on academic emotion recognition from artificial intelligence approach mainly focuses on the practical exploration of emotion recognition in the fields of natural language processing (Nandwani and Verma, 2021), computer vision, speech recognition, and physiological information recognition, mainly using text mining, speech recognition, and facial expression recognition, gesture recognition, biological information recognition and other technologies (Feng et al., 2020).

### 2.2. Emotion recognition based on text

Sentiment classification and analysis are important research front in the field of natural language processing, and emotion analysis is considered an extension and complement (Thelwall et al., 2012). Sentiment classification usually only focuses on the binary, whether it is positive or negative (Martín-Valdivia et al., 2013; Younis, 2021). However, according to emotional psychology research, although positive and negative are important emotional dimensions, there are still many other emotional types and emotional intensity measurement criteria, and positive and negative cannot meet the needs of emotional classification (Cornelius, 1996).

Both unsupervised and supervised approaches are used in research of emotion detection. Unsupervised approaches mainly including dictionary-based and rule-based methods. Mac Kim et al. (2010) used three datasets to build up an emotion word dictionary as a classification category, and classified the emotion texts into angry, fear, joy, and happiness. Agrawal and An (2012) added a discussion of syntactic relations based on emotional words, built a recognition model, and found that the accuracy rate has improved. Supervised approaches are mainly based on machine learning models (Asghar et al., 2019; Hasan et al., 2019; Tiwari et al., 2017), based on deep learning models or hybrid models (Basiri et al., 2020; Xu et al., 2020; Singh et al., 2021), and transfer learning techniques (Ahmad et al., 2020).

Research on academic emotions has been widely carried out, and many questionnaires are used for different research subjects. However, these methods are not suitable for academic emotion recognition process based on large-scale datasets. Meanwhile, the achievement of text-based emotion analysis, we can easily use these methods and techniques for academic emotion recognition research.

## 3. Data and methodology

### 3.1. Data collection

The dataset used in our research needs to meet two main requirements. First, to achieve opinion mining, it should be user generated content; Second, it should be posted by graduate students. We choose to collect the posts and comments from “Xiaomuchong”^1^, a famous Online Academic Forum in China, which focuses on provide a place for postgraduate students to exchange academic opinions, discuss study methods, and share daily lives. The forum has more than 5 million topics, 140 million comments and 25 million registered users. Besides the considerable amount of data capacity, the registration restrictions are critical, it requires postgraduate certification, and it is highly recommended to provide the registers major status. In this study, we focus on the academic emotions, so we need to make a preliminary limit to the data source just from the “postgraduate study mood” section.

We collected the web page text content of the Xiaomuchong website through a web spider program written in python for 2 weeks, and the data set contains three types of useful information: topic information, detail information and poster information. The topic information is the dataset of topics posted by the website users. It contains the title of the topic, the post time, the latest reply time, the id of the topic poster and the volume of views and replies. The detail information contains the whole detail content of the dataset. Each reply to every topic in our dataset is collected. It contains the replied detail text content and the replier’s information such as id and reply time. The poster information contains the personal information of both posters and repliers, such as age, gender and what they are majoring in. Finally, we collected 18,831 topic items, 511,269 detail items and 131,755 poster information items and stored in MongoDB.

### 3.2. Academic emotion recognition method

#### 3.2.1. Academic emotion recognition framework

**Figure 1:**
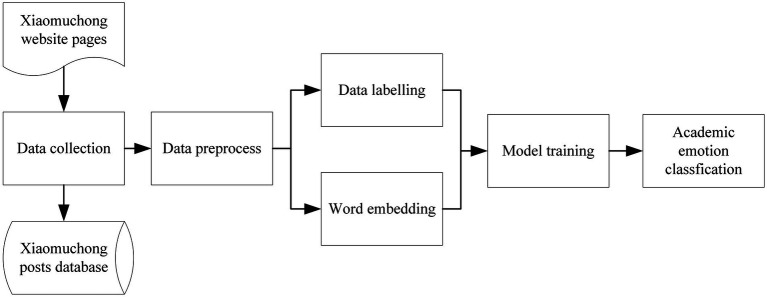
Academic emotion recognition data process pipeline.

We used Bi-GRU, a deep learning model, for training and testing to achieve the task of identifying academic emotions. The pipeline of the entire model includes three main steps, which is shown in Figure 1.

The first step is data pre-processing. For Chinese text, before the word vector embedding task, it is necessary to perform a word segmentation on the text. The second step is data labeling, it is essential in supervised learning model training. In the word embedding process, due to the necessity to train and test on different models and compare the results, we used a pretrained word2vec model instead of randomly initialized word vectors. We selected a Chinese word vector library developed by Tencent Artificial Intelligence Laboratory (Song et al., 2018). This library has more than 8 million Chinese words, and contains many emerging words and network terms, which is very suitable for machine learning or deep learning downstream tasks based on user generated content. After the word vector embedding step, the Chinese words after word segmentation can be converted into word vectors of 200 dimensions. The last step is model training. We split the dataset into training set and test set, and through multiple iterations of multiple epochs, we finally got a stable classification model and tested it on the test set.

#### 3.2.2. Data labelling

We labeled the dataset into four categories with academic emotion tags among “Positive activating,” “Positive deactivating,” “Negative activating” and “Negative deactivating.” Due to the large amount of data, perform manual labeling method on global data set is not appropriate. To ensure the efficiency and accuracy of data labeling result, we divide the process into two steps. The first round of labeling process is unsupervised labeling, and the second round is conducted by combining unsupervised and manual labeling. Unsupervised labeling is to determine whether the target text contains emotional words corresponding to the dimension. We first constructed an emotional word list based on academic emotion categories (Pekrun et al., 2011) and an existing Chinese emotional ontology (Xu et al., 2008), which contains the dimensions of academic emotion classification and the emotion words related to each dimension (As shown in Table 1). The ontology contains several attributes to each word, including lexicon, emotional category, and emotional strength.

**Table 1:** Academic emotion dimensions.

Academic emotion dimension		Emotional word categories (translated from Chinese)
Dimension	Sub-dimension	
Positive activating	Enjoyment, hope, joy	Happy, respect, praise, believe, love, wish
Positive deactivating	Relaxation	Relieved, comfort
Negative activating	Anger, anxiety, shame	Anger, fear, guilt, thinking, panic, shy, jealousy
Negative deactivating	Disappointment, boredom	Disappointment, sadness, bored, abomination, derogatory, doubt

Three conditions occurred during the process. First, there is only one emotional word in the text, which can be directly labeled with the corresponding dimension. Second, the text does not contain any emotional words, and it will be marked as invalid data. The third condition is the most complicated. If the text contains multiple emotional words, a method for calculating academic emotional strength was conducted to present the emotional tendency. For an unlabeled text, the academic emotional strength of one dimension can be calculated as the sum of the emotional strength of all emotional words belongs to this dimension, and the text can be labeled as the academic emotion dimension with the max emotional strength. For example, in an unlabeled text “*今天做完了实验，真开心, 但导师却挑剔的说结果不够好 (I’m really happy that I finished the experiment today, but my supervisor was critical and said that the result was not good enough)*,” there are two emotional word <*开心 (happy)*, *挑剔(critical)* > in this text which belongs two emotional word categories < *happy*, *derogatory* > and two academic emotion dimensions < *Positive activating*, *Negative deactivating* >. According to the emotional ontology, emotional strength of word “happy” is 7 and “critical” is 5, thus the academic emotion dimension strength of this text can be calculated as < *Positive activating:7, Positive deactivating: 0, Negative activating: 0, Negative deactivating:5* >, 7 is the max strength value, so this text will be labeled as “Positive activating.”

**Figure 2:**
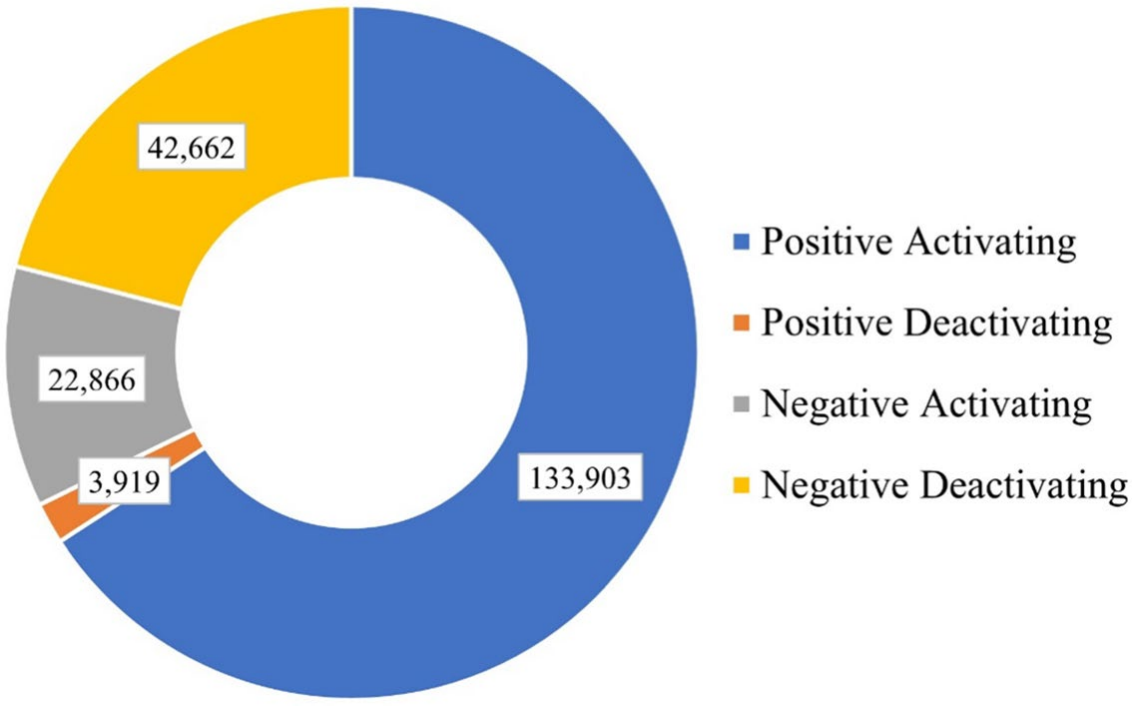
Academic emotion dimensions labeling distribution.

However, this unsupervised data labeling method is suitable for conditions where the text length should not be too long. If there are more than 10 emotional words in one unlabeled text, then it can be classified as long text (Deng and Ren, 2021). We manually checked these long texts, selected items with obvious emotional tendencies and marked them, and discarded other items. Finally, 203,350 detail items are labeled, and the distribution of each dimension of academic emotion are shown in Figure 2.

#### 3.2.3. BiGRU-attention model

From natural language processing aspect, the task of identifying academic sentiment from textual content can be considered as feature based text sequence classification, so among deep learning models and frameworks, a recurrent neural network (RNN) that can extract and process sequence features is conducted. GRU (Gated Recurrent Units) and LSTM (Long-short Term Memory) are both optimized recurrent neural network models (Chung et al., 2014), which are suitable for dealing with long sequences in the sequence model. Compared with the traditional RNN model, update mechanism has been added to enhance the memory ability and reduces the chances of gradient disappearance and gradient explosion. These two models both have a large scale of applications in the fields of text classification, machine translation, and speech recognition. As an improvement of the LSTM model, the GRU model simplifies the input gate and forget gate into one update gate in the unit of hidden layer (Yang et al., 2020). A gated recurrent unit contains an update gate *z*_t_ and a reset gate *r*_t_. At time *t*, according to the output of previous stateℎ *h*_t−1_ and current input *x*_t_, *z*_t_ and *r*_t_ can be formulated as:


$$z_{t}=\sigma \left(W_{z}\times \left[h_{t-1},x_{t}\right]\right)$$

$$r_{t}=\sigma \left(W_{r}\times \left[h_{t-1},x_{t}\right]\right)$$

After inputting the previous gated state, a reset gate can calculate an updated (*ℎ*_t−1_ × *x*_t_), then combine the new data with the current input *x*_t_, a gated recurrent unit calculates the new state *h*_t_ as:


$$h_{t}=\tanh\left(W_{r}\times \left[h_{t-1}\times r_{t},x_{t}\right]\right)$$

tanh is used as the activation function. This $h_{t}$ is the function of current input $x_{t}$ , also considered as the candidate hidden layer. $r_{t}$ can control the memory size, combines current $h_{t}$ and previous $h_{t-1}$ to calculate the final hidden layer state. Finally, the current $h_{t}$ calculation formular is updated as:


$$h_{t}=\left(1-z_{t}\right)\times h_{t-1}+z_{t}\times h_{t}$$

Here $z_{t}$ is the gate control signal, and $z_{t}\in \left[0,1\right]$ , it controls the forgetting amount of information. The closer the value is to 0, the more information needs to be forgotten.

**Figure 3:**
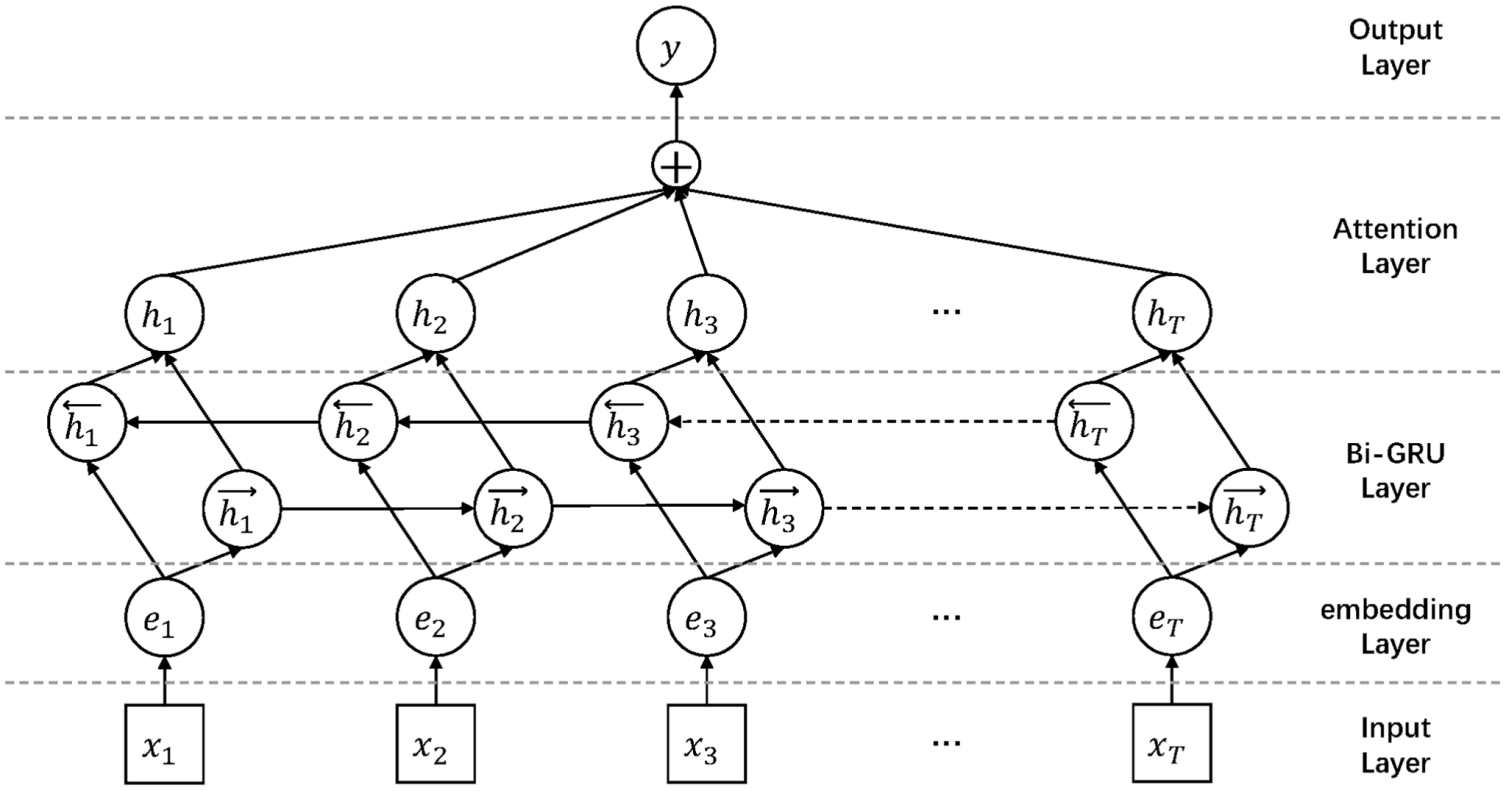
BiGRU-Attention classification model structure.

However, in sentiment classification tasks, use RNN model to extract text features has certain defects, that is, the one-way RNN model can only extract the former context features, but the latter context features are also very important. Using a bidirectional recurrent neural network structure can effectively solve this problem (Tang et al., 2019), and we conducted a bidirectional GRU network. The Bi-GRU model solves the sequence features in the text, and to achieve the classification function of the model, we need to adopt a classification structure. For the four classifications of the results, we employ a softmax classifier for concatenation. In addition, since we have performed word segmentation on the text, the importance of words will affect the classification results of the model, so an attention layer is added between the softmax layer and the hidden layer. The structure of the entire classification model is shown in the Figure 3.

Meanwhile, to figure out the performance of the model we adopted, we also used machine learning classification algorithm support vector machine, multinomial Naive Bayes and one-way GRU neural network as the baseline for comparison.

## 4. Results and discussion

### 4.1. Descriptive statistical analysis of posts

**Figure 4:**
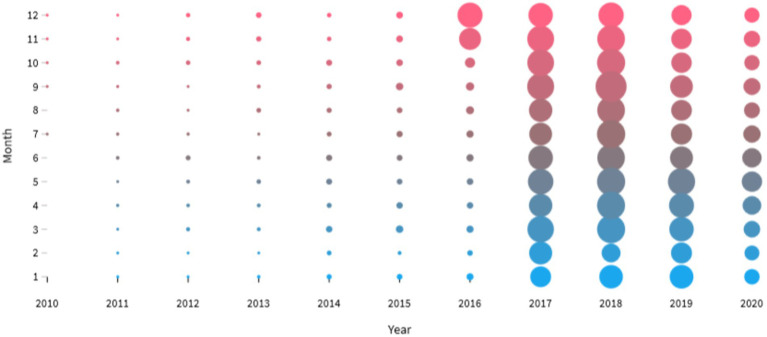
The year and month distribution of the topics.

The research data we have collected has been since the establishment of the Xiaomuchong website from 2010 to 2020. Finally, we collected 18,831 topic items, 511,269 detail items and 131,755 poster information items. The year and month distribution of topics is shown in Figure 4. From 2010 to 2015, these topics did not get much discussion, but after November 2016, the volume of topics increased dramatically and reached a peak around 2017 to 2019. Although in 2020 there was a small decrease in the volume of topics, but there is still a lot of attention on the topic of academic emotion among graduate students.

**Figure 5:**
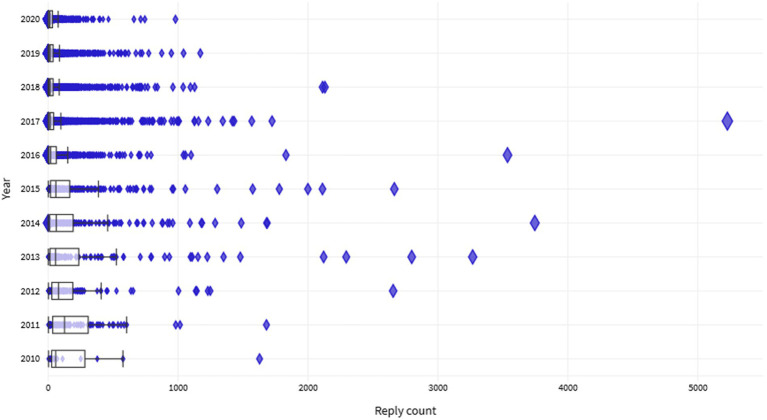
The distribution of the hot topics.

Meanwhile, to figure out which topics get the most discussion, we draw boxplots based on replies to those topics as shown in Figure 5. From the overall aspect, the mean and median of the volume of replies to the topics show the post time of the topic is related to the volume of replies. The earlier a topic is posted, the more exposure it gets, and therefore more replies are performed. Most topics received less than 1,000 replies, but there were also some topics that got more than 3,000 replies, with the most reaching an astounding 5,227, which is a topic about “PhD thesis defence successfully passed” posted in 2017.

### 4.2. Descriptive analysis of posters’ gender and major

To find out the academic emotions hidden in viewpoints from multiple perspectives, it is also very important to conduct statistical analysis on the direct data that can be obtained, such as the distribution of posters’ gender and major.

**Figure 6:**
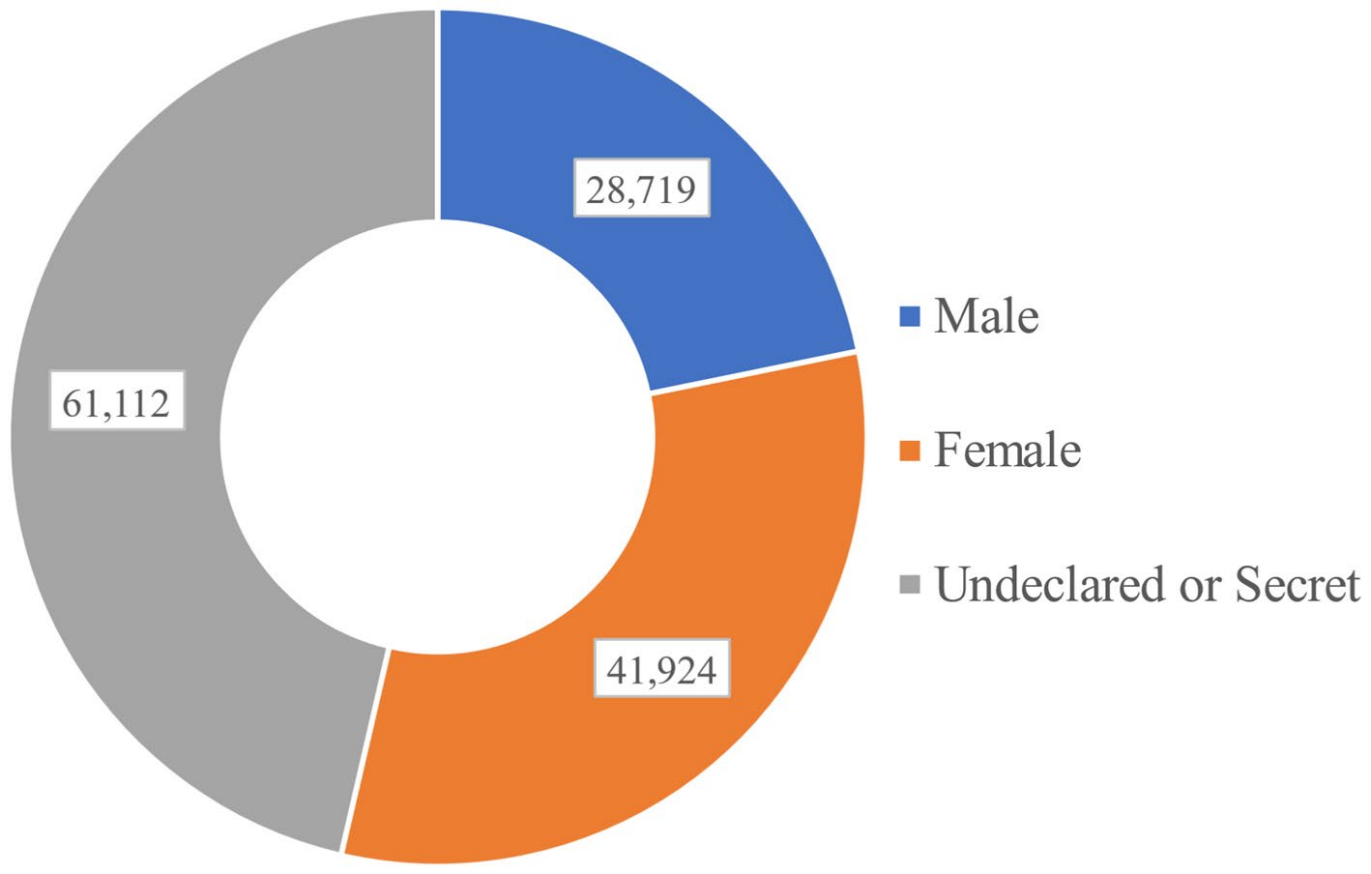
The gender distribution of the posters on Xiaomuchong.

The gender distribution of the posters is shown in the Figure 6. Surprisingly, nearly half of the posters (about 46.38%) chose not to set gender or keep gender confidential. When it comes to personal situations like sentiments and emotions, even in online communities that share opinions anonymously, people still tend to keep personal information such as gender in secret. Among the posters who set their genders, 21.80% of them selected as ‘Male’ and 31.82% of them selected as ‘Female’. In such online communities, female graduate students are more willing to express their thoughts and opinions.

**Figure 7:**
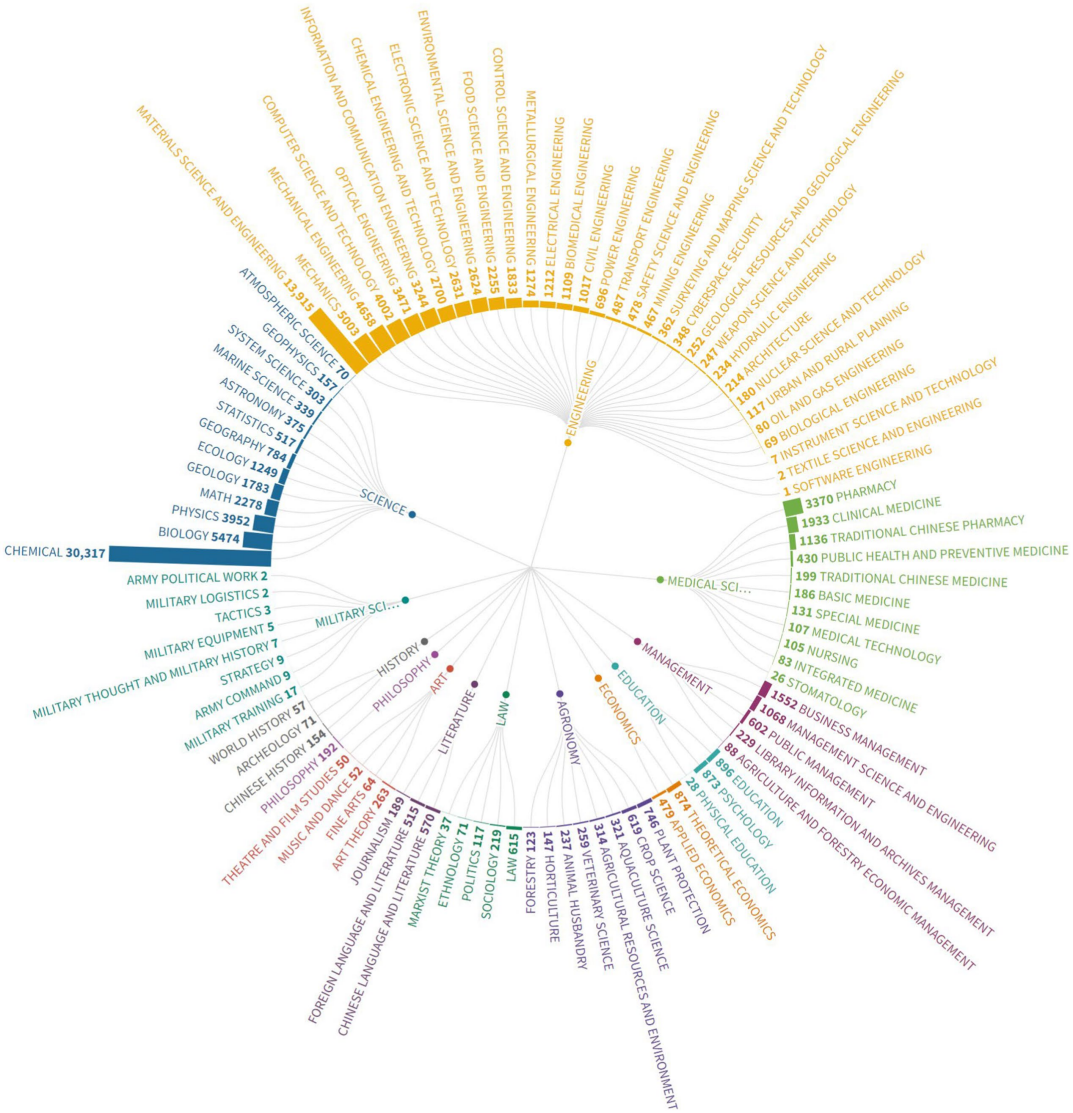
The major distribution of the posters on Xiaomuchong.

As a graduate student, different majors have different academic pressures. For example, the students majoring in engineering may suffer from the unsatisfactory optimization of the experimental model and not get enough ideal experimental data, Meanwhile, the students majoring in literature might suffer from lack of inspiration and creation of new chapters. It is important to figure out the distribution of the posters’ majors. However, it is inappropriate to show the distributions directly because there are too many different majors, and some majors are quite alike. So, we classified the majors into 13 main subjects and 111 subcategories manually according to *Catalogue of Degree Granting and Talent Training Subjects* published by the Ministry of Education of the People’s Republic of China.^2^ At last, we conducted a statistical analysis of the subjects of the posters’ majors, and the result is show in Figure 7.

We can find out that the volume of posters on online platforms varies widely among graduate students in different majors. From the main subject level, the “engineering” subject not only has the largest total volume of posters, but also has the most kinds of subcategories. Due to the sensitivity and the quantity of samples collected, the volume of posters belonging to military science is the least, with no more than 100 records. Obviously, the difference between the majors of the article is more significant from the subcategory level. Among the graduate students who post on the Xiaomuchong website, the students majoring in “chemical” are the most, reaching 30,317. This is not an accidental phenomenon and there have been many conclusions about the academic pressure and academic depression of graduate students majoring in chemistry (Rodrigues, 2020; Stockard et al., 2021). Meanwhile, the second place is the students majoring in “material science and engineering” with 13,915. These are the only two majors with over 10,000 records. Is this phenomenon because the students of these two majors are more inclined to express their opinions and views online? Or do they have more academic emotions to express? We found some valuable reasons from other literature (Tang et al., 2018; Woolston and O'Meara, 2019). One of the main reasons is that students in these two majors, especially graduate students, face greater academic pressure. The daily work of these graduate students in these two majors is based on a giant number of experiments. A lot of work means extra working hours, which leads to a lack of work-life balance, anxiety, and depression.

### 4.3. Result of academic emotion classification

We randomly selected 203,350 data records to obtain the training set at a ratio of 80% and rounded them to integers. The training set contains 160,000 data records, and the remaining 43,350 are used as the test set.

In the performance evaluation of the classification model, we mainly carried out two aspects. The first is to measure the overall model performance, using overall indicators for evaluation. Then, to discover the classification effect of each category, we also evaluated the precision, recall and F1score of each category. For the overall metrics, we selected macro average score for evaluation. The results are shown in Table 2 (PA: Positive activating, PD: Positive deactivating, NA: Negative activating, ND: Negative deactivating).

**Table 2:** Performance comparison for each model.

Model	Indicators	Overall (Macro average)	Categories			
			PA	PD	NA	ND
BiGRU-attention	Precision	0.97	0.98	0.96	0.98	0.95
	Recall	0.95	0.99	0.89	0.96	0.96
	F1	0.96	0.98	0.93	0.97	0.98
GRU	Precision	0.85	0.86	0.84	0.86	0.83
	Recall	0.83	0.84	0.82	0.84	0.81
	F1	0.82	0.83	0.81	0.83	0.80
Multinomial NB	Precision	0.65	0.66	0.64	0.66	0.63
	Recall	0.64	0.65	0.63	0.65	0.62
	F1	0.62	0.63	0.61	0.63	0.60
SVM	Precision	0.72	0.73	0.71	0.73	0.7
	Recall	0.70	0.71	0.69	0.71	0.68
	F1	0.71	0.72	0.70	0.72	0.69

Comparing between different models, deep learning models perform better than machine learning models. We can find that the BiGRU-Attention model we used has the best scores on various indicators all above 95%, shows that the model has good performance. The comprehensive score of the one-way GRU model has also reached more than 80%, which is also fair enough. However, performance of the machine learning models, multinomial NB and svm, are both unsatisfactory. The svm model can barely reach 70% in all three indicators, while the naive Bayes model performs even worse, only slightly more than 60%.

Furthermore, we can find that the classification model has good performance on all four categories. The PA (Positive Activating) classification with the best effect has a score of more than 98% in all three indicators. This category has the largest sample size and a larger range of emotional words, which can have better recognition effect. The worst result of the four categories is PD (Positive Deactivating), with a F1 score of 93% and a recall 89%. Just focus on the score of this one category, the performance of the model is good enough, but there is a gap of close to 5 to 10% with the scores of the other three categories. This phenomenon occurs because this category PD (Positive Deactivating) is one of the academic emotions which is most difficult to articulate or to define. According to Pekrun et al. (2011)’s research, academic emotions in the category of PD (Positive Deactivating) are considered to have positive sentiment, but a negative effect on academic efforts. For example, if a graduate student completes light work with ample time constraints, he will perceive it as an “easy” task and will feel “relieved” and “comfortable.” Then such emotions will have a negative effect on the following work, which may lead graduate students to think that research and learning are very simple, which is not conducive to their concentration on tasks. But somehow the emotional words of are very close to the PA (Positive Activating) category. Thus, “happy” and “relieve” are very easy to distinguish, but it is difficult to define whether “satisfaction” will have a positive or negative impact on academic efforts. This is the main reason for the low recall rate of this category.

In general, in such a high-dimensional multi-classification natural language processing task, using a deep learning model based on a recurrent neural network combined with an attention mechanism to build a classification model can achieve good performance with more than 90% of the comprehensive scores. The performance indicators can support some Chinese text-based academic emotion recognition applications based on such a model.

### 4.4. Topic analysis of graduate students’ emotional engagement

Discovering hidden academic emotions from posters can effectively help us deal with the academic stress of graduate students, but it is not enough to know that what are they really talking about or worrying about. To have a further understanding of current graduate students using online platforms, it is necessary to bring up this discussion to semantic and topic level.

**Figure 8:**
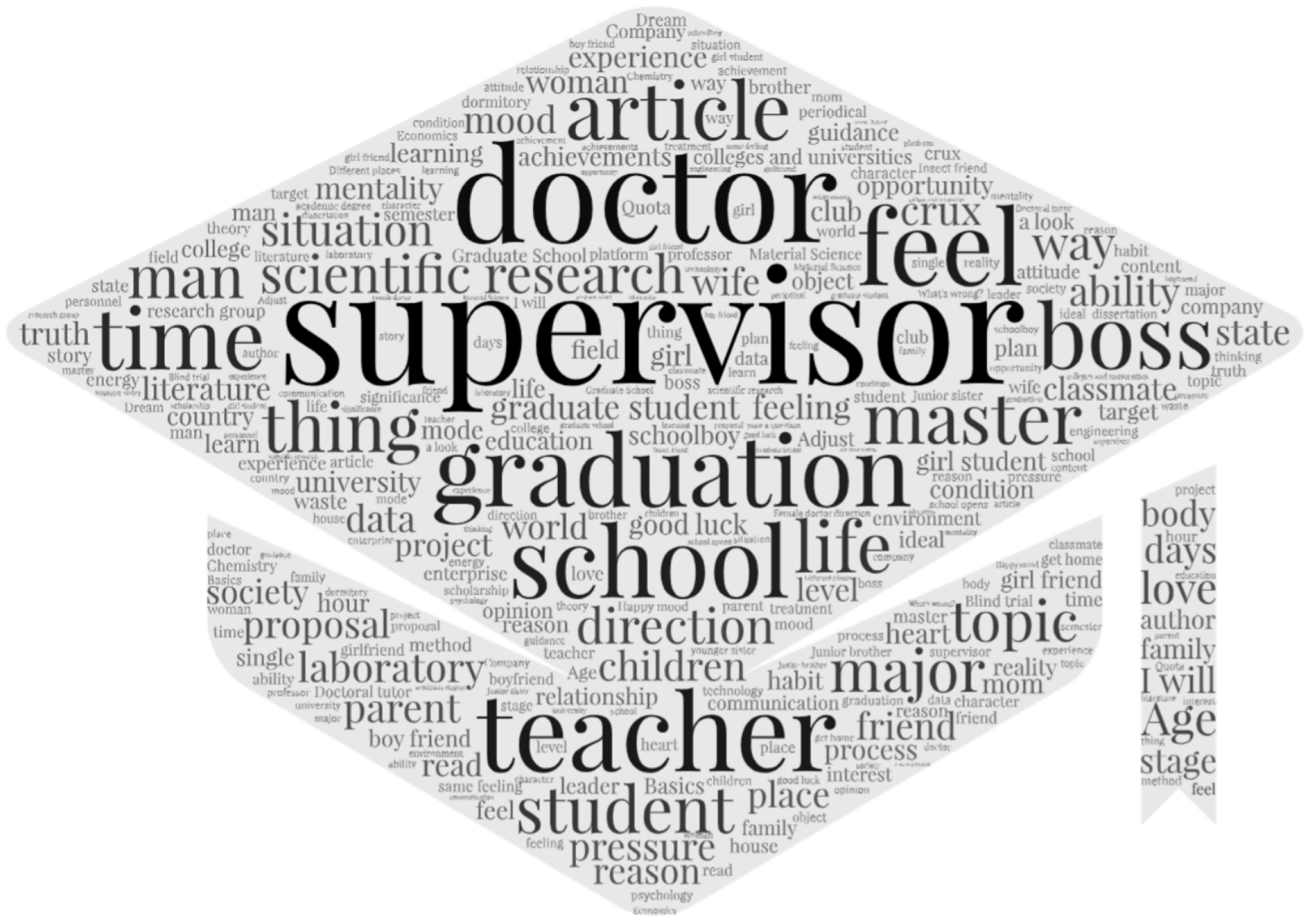
High frequency words word cloud. *N*_word_freq_ > 100, translated.

In addition to identifying the hidden graduate academic emotions from the website’s posts, to truly understand the specific topics that the graduates are discussing, we have carried out more detailed discussions and analysis. After the text pre-processing operation, we not only construct the recognition model of the obtained corpus, but also conduct a simple topic analysis on the collected topics. Through word frequency statistics, we excluded keywords with term frequency less than 1,000, finally, we can know high-frequency words as shown in Figure 8.

We continue to use the Chinese word vectors obtained in the word vector embedding step and conducted a k-means algorithm to cluster the words in each topic and post, determined k=8 by calculating the silhouette coefficient, and finally divided the results into eight categories unsupervised. Based on these eight clusters, we invited 3 experts in postgraduate admissions employment and postgraduate mental health to summarize and manually classify them to improve the readability of the results. Finally, we divided these subject headings into the following three categories: academic pressure related topics, interpersonal related topics, and career related topics.

#### 4.4.1. Academic pressure related topics

Academic pressure is a major aspect of postgraduate students expressing academic emotions. The academic emotional pressure of postgraduates is not only good or bad academic exam score performance, but also includes the pressure of scientific research achievements, such as experiments and papers. The high-frequency words related to the academic pressure of graduate students include “papers,” “projects,” “laboratories,” and “scientific research,” etc. Academic pressure, that is, the dual pressure from study and scientific research, is the topic that postgraduates are most concerned about, and may also be the major source of postgraduate pressure. Accompanied by such academic pressure, psychological problems or practical problems may occur among graduate students. Psychological problems can lead to mental illness, such as depression, obsessive–compulsive disorder, anxiety disorder, and even lead to schizophrenia, etc. (O'Connor and Yanos, 2021). Suicidal behavior is not uncommon for master and doctoral students (Poreddi et al., 2021). On the other hand, practical problems are mainly focused on “how to graduate.” In China’s postgraduate training program, the number of scientific research achievements of postgraduates within the school duration is closely related to whether they can successfully graduate and obtaining a graduation certificate and a degree certificate. Therefore, for graduate students, publishing academic papers and conducting scientific research is not only the accumulation of their own interests or enthusiasm, but also a necessary factor to ensure that they can successfully graduate.

#### 4.4.2. Interpersonal related topics

Another important emotion topic comes from lack of social interaction. The high-intensity research and study of graduate students makes their life trajectory very simple. The daily life of most graduate students during the semester is to commute among the laboratory or classroom, dormitory and canteen. They do not have the opportunity, nor the extra energy, to engage in social activities. This situation is more prominent among Chinese students (Moore-Jones, 2022). The high-frequency words related to the interpersonal pressure of graduate students include “friends,” “relationships,” “lovers,” and “families,” etc. The lack of social activities and the unmet need for friendship are mutually influencing and sometimes reinforcing (Suwinyattichaiporn and Johnson, 2022). Somehow, the need for a spouse or couple cannot be ignored in the social emotions of graduate students. Keywords like couples, boyfriends and girlfriends, and marriage also have high word frequencies. Among these posts, many of them are marriage and friendship posts. For graduate students who are socially deficient, this may be a relatively effective way for them to be more familiar with, or to hope for O’Day and Heimberg (2021). There is also a certain number of posts discussing family and relationships. These discussions are not the same as those about finding the other half, which are mostly about the relationship between graduates themself and their parents. According to China’s higher education system, graduate students at the master’s level are usually around 25 years old, and those at the doctoral level are around 27–30 years old. At this age, the relationship with parents is at a low standard in lifetime (Kremer, 2016). There is a significant work–family conflict (WFC) between graduate students and their parents (Dolson and Deemer, 2022), which can be simply summarized as the income level of graduate students is incomparable with work, parents need to continue to provide help for their children’s lives, and the material and mental pressure that children bear in this relationship as graduates are seriously facing.

#### 4.4.3. Career related topics

Finally, one of the most frequently discussed topics online for graduate students is related to personal career development. These topics are like the WFC mentioned in the previous section, but more about the concerns of the graduate students themselves about their personal career development. The high-frequency words related to the career pressure of graduate students include “work,” “career,” “job,” and “income,” etc. These pressures and emotions arise from comparison. We all know that academic qualifications and degrees can determine the type and starting point of your future work, but in actual working environment, personal career development is often influenced by a variety of factors, among which working years is a very important consideration (Tang et al., 2008; Purohit et al., 2020). This brings up the first comparison, the comparison between peers. Compared to their peers, if they did not continue to study for a master’s or doctoral degree but started working after graduating from an undergraduate or junior college, their income level is likely to be higher than that of fresh graduate students due to the accumulation of working years (Yusuf et al., 2020). Another comparison is with one’s own efforts. Pursuing a master’s degree can take 3 years or more, and a doctorate requires at least 6–7 years of additional study time compared to a bachelor’s degree. It is really a matter of willpower and endurance, and a lot of mental work. But the input–output ratio of a graduate student’s first job is often suboptimal, the value created, and the income obtained may not be satisfied with what the graduate student thinks is equivalent. These two comparisons are very easy to make graduate students have a psychological gap and self-doubt, thinking that their efforts are worthless, have no prospects, and have not improved their living standard. Not only in China, but many graduate students all over the world have the same worries and anxieties (Are et al., 2018; McConnell et al., 2018; Sharif et al., 2019).

## 5. Conclusion

Measuring academic emotions is an important way to discover graduate students’ learning status and mental health. Due to the concealment and diversity of academic emotions, it is difficult to discover hidden them from texts using traditional methods. The academic emotions and academic pressures of graduate students is a long-standing concern and is receiving increasing attention. The peculiarity of graduates’ academic emotions is that their stress sources are not only from their studies, but also from research, family, and career planning. At the same time, with these academic pressures, there is no effective way to express and talk about these academic emotions that graduate students generate. In the long run, it is very easy to form psychological problems and lead to serious consequences. Many studies have analyzed and excavated academic emotion.

To address the three research questions we raised, we conducted a series of academic emotion recognition and analysis methods on large-scale datasets. For our first research question, our research conducted a statistic analysis of the collected postgraduates’ posts on Xiaomuchong platform, mainly gender and major, and find out excludes users who do not want to disclose their gender or who do not fill in their gender, female post more on the platform. According to the majors marked by posters, we found that graduate students in science and engineering published most of the posts on the platform, especially majoring in chemistry. This is determined by the features of the major and the way in which the research work undertaken is carried out. For our second research question, we transform the academic emotion recognition task into a series process of constructing, training, and testing an emotion classification model based on user-generated text content. Aiming at the shortcomings of traditional academic emotion recognition research in the application of large-scale data sets, we constructed a pipeline based on recurrent neural network, which can identify and classify academic emotions unsupervised, and has a relatively ideal model performance. At last, for our third research question, based on the word vectors, we performed a topic analysis among the graduate students’ posts. We clustered graduate posts on the Xiaomuchong platform into three main categories: academic pressure related topics, interpersonal related topics, and career related topics. We also discussed the main problems and sources of stress faced by graduate students from these three main categories. There are also deficiencies in our research that need to be improved in future research. The first is the problem of data labeling. The use of vocabulary-based heuristic rules may be insufficient. Consider using a decision tree model instead. Second, the topic of posts is not necessarily a simple emotional expression, but also a relevant topic discussion. The information organization provided only through the website may be insufficient. Consider a better way to filter the data set.

## Data availability statement

The raw data supporting the conclusions of this article will be made available by the authors, without undue reservation.

## Author contributions

QX: research design and paper writing. SC: data collection and data analysis. YX: paper revision and editing. CM: paper writing. All authors contributed to the article and approved the submitted version.

## Funding

This work is supported by the National Natural Science Foundation of China (Nos. 72104219 and 62207016), the MOE Project of Humanities and Social Sciences (No. 21YJC870013), and Major Humanities and Social Sciences Research Projects in Zhejiang higher education institutions (No. 2023QN129).

## Conflict of interest

The authors declare that the research was conducted in the absence of any commercial or financial relationships that could be construed as a potential conflict of interest.

## Publisher’s note

All claims expressed in this article are solely those of the authors and do not necessarily represent those of their affiliated organizations, or those of the publisher, the editors and the reviewers. Any product that may be evaluated in this article, or claim that may be made by its manufacturer, is not guaranteed or endorsed by the publisher.

## Footnotes

^1^A forum for academic information exchange among researchers. http://muchong.com/bbs/

^2^The Catalogue is according to the version published in 2018 by MOE. http://www.moe.gov.cn/s78/A22/xwb_left/moe_833/201804/t20180419_333655.html
